# Use of allogenous bone graft and osteosynthetic stabilization in treatment of massive post-sternotomy defects

**DOI:** 10.1186/1749-8090-8-S1-P70

**Published:** 2013-09-11

**Authors:** M Kalab, B Kubesova, V Lonsky

**Affiliations:** 1 Department of Cardiac Surgery, University Hospital and Faculty of Medicine, Palacky University, Czech Republic; 2 National Tissue Centre, Brno, Czech Republic

## Background

Transverse plate fixation of the sternum is a currently used method for treatment of sternal dehiscences. Application of transverse titanium plates enables safe restoration of the chest wall stability. However, a massive deficiency in bone tissue of sternum and neighboring ribs often places limits to its use.

## Methods

Capitalizing on orthopedic surgery experience, in 6 cases we replaced a massive deficiency of chest skeleton by an allogenous bone graft and applied transverse plate osteosynthesis to achieve stabilization of bone grafts and chest wall. We have used an allogenous bone grafts of sternum prepared by the tissue bank according to Czech legislation. In treatment of an early infection of the wound, we usually apply the vacuum assisted drainage to achieve 2 subsequent negative results within microbiological check. Before bone grafts implantation, we always performed prophylactic resection of the residual skeleton edges 1 or 2 cm to the healthy bone tissue. Transverse plate fixation of the chest wall was applied. Line of contact between the graft and osseous fragments of the patient were filled with spongious bone tissue prepared from an allogenous graft of the femur.

## Results

A check-up clinical examination and CT examination 6 months later showed excellent results in 5 cases. In one case, the patient died of hypostatic bronchopneumonia 2 months after the graft implantation. However, an autopsy proved the wound completely healed with no marks of local infection. In the first case, a calve bone graft was applied, and sternal bone grafts were applied in the five following cases.

## Conclusions

Allogenous bone graft transplantation could be a novel approach to achievement of maximum stability of the chest wall in the management of complicated sternal dehiscence. In our experience, two principles are paramount: early radical tissue debridement and strict repeated microbial examination of osseous fragments of the wound.

